# Farmers' Perception of the Health Effects of Agrochemicals in Southeast Nigeria

**DOI:** 10.5696/2156-9614-8.19.180901

**Published:** 2018-08-20

**Authors:** Chikamso C. Apeh

**Affiliations:** African Heritage Institution, Enugu, Nigeria

**Keywords:** agrochemicals, farmers, health, Nigeria

## Abstract

**Background.:**

Agrochemicals are used by farmers in Southeast Nigeria to increase crop yields and food production. However, farmers are often illiterate and do not follow precautions for their usage and application, increasing the risk of exposures to humans and the environment.

**Objectives.:**

The aim of the present study was to determine the extent of the use of agrochemicals by farmers, category or type used, ability to read instructions, exposure to agrochemicals during application and perception of the health effects of exposure to agrochemicals in Southeast Nigeria.

**Methods.:**

From February–June 2017, a total of 200 farmers were surveyed using oral interviews and structured questionnaires. Data were analyzed using descriptive statistics. Respondents were asked about items such as socioeconomic characteristics, types and amounts of fertilizers and pesticides used, exposure during application and perceptions of the health effects of exposure to agrochemicals.

**Results.:**

In the present study, the majority of farmers (74%) used inorganic fertilizers (nitrogen, phosphorus, and potassium (NPK)) and 26% used organic fertilizers (compost manure). Most of the farmers in the present study (65%) reported that they could not read agrochemical application instructions, 92% of farmers were exposed to agrochemicals during application, and most farmers (73%) reported falling sick after exposure to agrochemicals.

**Conclusions.:**

We recommend that agricultural extension agents provide farmers with comprehensive training in agrochemical use to ensure their health and lower environmental risks.

**Participant Consent.:**

Obtained

**Ethics Approval.:**

The study was approved by the Research Ethics Committee, Department of Agricultural Economics, Faculty of Agriculture, University of Nigeria, Nsukka

**Competing Interests.:**

The author declares no competing financial interests

## Introduction

Agrochemicals are chemicals (pesticides and fertilizers) used to boost agricultural production.^1^,^2^ They are used as soil conditioners, acidifiers, nutrients and are also used to control diseases caused by bacteria, fungi, pests and viruses, enhancing agricultural productivity and safety. Factors such as balanced use, optimum dosing, correct application methods and timing help ensure increased agricultural productivity.^3^ Use of agrochemicals has led to increased food production.^4^,^5^ However, exposures to other organisms during their application, including humans, is poorly controlled.^6^ Their use has significantly increased the concentration of toxic materials in food and the environment, with negative effects on plant and animal health.^7^ The World Health Organization (WHO) has estimated that more than three million farmers in developing countries are poisoned by agrochemicals each year.^8^

In Nigeria, the agricultural sector is the major supplier of food, raw materials and foreign exchange, and 70% of Nigeria's population largely depends on this sector for survival.^9^,^10^ Due to the country's drive to increase agricultural production and the upsurge of different species of pests that damage and ravage agricultural products in fields and storage, farmers have resorted to the use of agrochemicals as an important control strategy.^11^ An estimated 125,000–130,000 metric tons of pesticides are used annually.^12^ According to Rahman and Chima, 70% of rice and yam farmers apply pesticides, and 41% of farmers apply pesticides to at least one food crop in Nigeria.^13^

The application of agrochemicals is often imprecise, with unintended worker exposures. A review by Asogwa and Dongo of problems associated with pesticide usage and application in cocoa production in southern Nigeria found that use of pesticides for pest control has generated public health problems and environmental pollution in Nigeria.^12^ Oruonye and Okrikata found that Nigeria's drive to boost food security and fight off insect pests and yield-limiting crop pathogens has led to unintended consequences such as mass importation and build-up of obsolete and toxic pesticides, with serious negative impacts on the ecosystem.^14^ A survey in 2012 found that although most Nigerians (74%) are aware of the European Union (EU) pronouncement on pesticide use, some of the banned chemicals (aldrin, binapacryl, captafol, chlordane, chlordimeform, DDT (dichlorodiphenyltrichloroethane), dieldrin, dinoseb, ethylene dichloride, heptaclor and lindane, parathion, phosphamidon, monocroptophos, methamidophos, chlorobenzilate, toxaphene, endrin, merix endosulphan, delta HCH (hexachlorocyclohexane) and ethylene oxide) are still being used by farmers.^15^,^16^ Abubakar et al. found that 93.8% of vegetable farmers along the Ngadda River in Nigeria applied pesticides, and most (85.2%) did not use protective clothing when doing so, exposing themselves to the adverse health effects of pesticides.^17^ Okafoagu et al. reported that 83% of farmers using agrochemicals exhibited unsafe practices (eating food while spraying, home storage of empty pesticide cans and using empty pesticide bottles for home water storage) in Sokoto, northwestern Nigeria.^18^

Currently, many agrochemical users in Nigeria are not properly informed of the risks and precautions involved in the application of toxic chemicals.^6^ Few studies have been conducted on agrochemical use in Nigeria, and only one in Southeast Nigeria.^11–15^,^17–21^ Studies showed evidence of a relationship between farmers' exposure to agrochemicals and illiteracy. Due to the toxic hazardous nature of agrochemicals, it is crucial that measures be taken to ensure the safety of farmers.^22^ So far, no study has been carried out to examine farmers' perception of the health effects of the use of agrochemical in Southeast Nigeria. Therefore, the aim of the present study was to determine the extent of the use of agrochemicals by farmers, category or type used, ability to read instructions, exposure to agrochemicals during application and perception of the health effects of exposure to agrochemicals in Southeast Nigeria. This will be helpful in the monitoring of agrochemical use and serve as a policy instrument for improved farming and ensuring the health of farmers in this region.

## Methods

The present study was conducted in Southeast Nigeria, an area comprised of five states: Imo, Anambra, Abia, Enugu and Ebonyi. This region, which is in the tropical rainforest zone of Nigeria, ranges from 4° 30′ to 7° 00′ N latitude and 5° 30′ to 9° 30′ longitude. The population of the study area was 31,371,941. Because of the dense population of this area (416 persons/km^2^), people there depend heavily on land resources.^23^

The present study was conducted from February–June 2017. A total of 200 farmers were surveyed, using oral interviews and structured questionnaires. Multi-stage random sampling was employed. First, four agrarian local government areas were purposively selected in each of the five states (20 local government areas selected), and then 10 farmers were randomly selected from each of these 20 local government areas (200 farmers selected). Poor funding was the only limiting factor to the number of farmers surveyed. The content of the instrument was validated by two experts from the Department of Agriculture, University of Nigeria Nsukka. Each was given a draft copy of the questionnaire accompanied by the specific research objectives. Their observations and comments were used in the correction of the final copies of the instruments. The questionnaire was administered to the literate farmers, while the researchers interviewed the illiterate farmers guided by the content of the questionnaire and their responses were recorded accordingly to ensure the accuracy of collected data. Respondents gave their consent by signing the consent form attached to the questionnaire. The study was approved by the Research Ethics Committee, Department of Agricultural Economics, University of Nigeria Nsukka.

Data were analyzed using descriptive statistics. Respondents were asked about items such as socioeconomic characteristics, types and amounts of fertilizers and pesticides used, exposure during application and perceptions of the health effects of exposure to agrochemicals.

## Results

The results in Table 1 show that 65% of farmers surveyed were male, and most were married (74%), with a mean age of 42 years (range: 23 to 80 years). On average, farmers had 11 years of education (range: 0 to 19 years) and 15 years of farming experience (range: 0 to 47 years). The average household size was 6 people (range: 1 to 19 people), and average farm size was 3 hectares (range: 1 to 6 hectares).

**Table 1:** Socioeconomic Characteristics of Respondents

**Variable**	**Mean**	**Min**	**Max**
Age (Years)	42.11	23	80
Gender	0.65 (Male = 65%, Female = 35%)	0	1
Marital status	0.74 (Married = 74%, Unmarried = 26%)	0	1
Education (Years)	11.20	0	19
Years farming	15.01	0	47
Household size	6.31	1	19
Farm size (Hectares)	03.03	1	6

This represents the seven socioeconomic characteristics of respondents studied. Age ranged from 23 to 80 years. Level of education ranged from 0 to 19 years. Farming experience ranged from 0 to 47 years. Household size ranged from l to 19 and farm size ranged from 1 to 6 hectares.

This represents the seven socioeconomic characteristics of respondents studied. Age ranged from 23 to 80 years. Level of education ranged from 0 to 19 years. Farming experience ranged from 0 to 47 years. Household size ranged from 1 to 19 and farm size ranged from 1 to 6 hectares.

Table 2 shows that the majority (85%) of farmers use agrochemicals, similar to the finding of Bassi et al. that 96% of the sampled farmers used agrochemicals in the Fadan-Daji district of Kagoro Chiefdom, Kaura Local Government Area, Kaduna state, Nigeria.^24^ In the present study, the majority (74%) used inorganic fertilizers (nitrogen, phosphorus, and potassium (NPK)) and 26% used organic fertilizers (compost manure). Most of the farmers in the present study (65%) could not read agrochemical application instructions, 92% of farmers were exposed to agrochemicals during application, and most farmers (73%) reported falling sick after exposure to agrochemicals.

**Table 2:** Agrochemical Use and Farmers' Perception of the Health Effects of Exposure to Agrochemicals

**Variable**	**Frequency**	**Percent (%)**
**Level of Agrochemical use**		
Use agrochemicals	170	85
Do not use agrochemicals	30	15
**Total**	**200**	**100**
**Type of fertilizer used**		
Organic fertilizer	052	26
Inorganic fertilizer	148	74
**Total**	**200**	**100**
**Level of pesticide use**		
Use pesticides	142	71
Do not use pesticides	58	29
**Total**	**200**	**100**
**Ability to read agrochemical instructions**		
Able to read	070	35
Unable to read	130	65
**Total**	**200**	**100**
**Farmers exposure to agrochemicals during application**		
Exposed	184	92
Not exposed	016	08
**Total**	**200**	**100**
**Farmer's perception of the health effects of exposure to agrochemicals**		
Sick	145	72.50
Not sick	039	19.50
Indifference	016	08.00
**Total**	**200**	**100**

## Discussion

The demographic characteristics presented in Table 1 indicate that farmers in the study area are largely young and of working age. This supports the findings of Omorogbee et al. in a study of farmers in Nigeria.^25^,^26^ They tended to have a poor educational background, many years of farming experience and the majority were married, with many household members to feed.^26–28^ These circumstances seem likely to have increased farmers' desire to adopt innovations to enhance production, and produce surplus crops to sell for profit. Farmers with more experience are more likely to use pesticides.^13^ The present study also supports the findings of Bassi et al. and Eifediyi et al. that more men than women are involved in farming in Nigeria.^24^,^29^

Of the farmers surveyed, 170 (85%) reported using agrochemicals, presumably to maximize production. This is supported by the findings of Bassi et al. and Eifediyi et al. that 96% and 65% of farmers use agrochemicals, respectively, in their farming.^24^,^29^ All of the farmers reported using fertilizers; 148 (74%) said they used inorganic fertilizers (NPK), and 52 (26%) said they used organic fertilizers (compost manure). Inorganic fertilizers were widely used by both educated and uneducated farmers, and 22 (11%) had more than 11 years education, while 178 (89%) had 11 years or less of education. Possible reasons for the low use of organic fertilizer may have included lower productivity, risk aversion, and cultural reasons. Abubakar et al. reported that nonorganic farming yields more than organic farming when compared in developing countries.^17^ Among farmers, 142 (71%) reported using pesticides for the control of weeds and insects to enhance productivity.

In total, 130 of the farmers (65%) were unable to read the instructions for the use of agrochemicals. This finding is consistent with those in a 1990 WHO study cited in Erhunmwunse that found that pesticide application problems arose due to farmers' inability to read and poor awareness, and two additional studies^30^,^31^ reported that farmers' illiteracy/inability to read information written on pesticides labels is largely responsible for their improper handling of pesticides.^19^ Inability to read instructions increases the likelihood of agrochemical misuse and thus of hazards to humans and the environment. According to Zare et al., the high rate of farmer illiteracy leads to their lack of knowledge about the side effects of pesticides and methods to alleviate these side effects.^32^

Of the farmers studied, 184 (92%) were exposed to agrochemicals during application. This is consistent with the findings of Abubakar et al. who found that 93.8% of the farmers along the Ngadda River in Maiduguri, Nigeria applied pesticides in their farming and 85.2% did not use protective clothing.^17^

Among the farmers, 145 (73%) reported having fallen sick after exposure to agrochemicals. In oral interviews, some recounted that they felt dizzy and depressed after exposure to agrochemicals and said that diagnosis in the hospital indicated that exposure was the cause. This finding is in line with those of previous studies that found that exposure to agrochemicals caused various symptoms such as mood disorders, depression, and anxiety, which were assumed to result from changes in the function of the nervous system.^12^,^33–37^

## Conclusions

The present study examined farmers' perceptions of the health effects of the use of agrochemicals in Southeast Nigeria. The study found that agrochemicals were used by 85% of the farmers in the study area to maximize productivity. In addition, 74% and 71% of farmers used inorganic fertilizers and pesticides, respectively, to enhance production. Most of the farmers (65%) had low levels of education and were unable to read instructions for agrochemical use, therefore increasing the likelihood of agrochemical misuse and exposure to farmers. In total, 92% of farmers were exposed to the harmful effects of agrochemicals during their application. Finally, approximately 73% of farmers studied reported haven fallen sick after exposure to agrochemicals. We recommend that agricultural extension agents provide farmers with comprehensive training in agrochemical use to ensure their health and lower environmental risks.
